# A Systematic Review of Household and Family Alcohol Use and Childhood Neurodevelopmental Outcomes in Low- and Middle-Income Countries

**DOI:** 10.1007/s10578-020-01112-3

**Published:** 2020-12-28

**Authors:** Tausif Huq, Emma C. Alexander, Logan Manikam, Tahir Jokinen, Priyanka Patil, Darrin Benjumea, Ishani Das, Leslie L. Davidson

**Affiliations:** 1 GKT School of Medical Education, King’s College London, London, UK; 2 Paediatric Liver, GI and Nutrition Centre and Mowatlabs, King’s College Hospital, London, UK; 3 Aceso Global Health Consultants Limited, London, UK; 4 UCL Institute of Epidemiology and Health Care, University College London, London, WC1E 7HB UK; 5 Mailman School of Public Health, Columbia University, New York, USA

**Keywords:** Alcohol, Alcohol abuse, Low- and-middle income countries, Neurodevelopment, Behaviour problems, Children

## Abstract

Childhood exposure to alcohol misuse by household adults has been related to childhood developmental delay, cognitive impacts, mental illness, and problem behaviours. Most evidence comes from high income countries. This systematic review only included studies from low- and middle-income countries (LMICs). Five databases were searched from 1990–2020. Twenty-eight studies of children 0–12 years were included, with 42,599 participants from 11 LMICs. The most common outcome was behavioural problems/disorders (19 studies). Despite varying study designs, this review found that alcohol misuse by household members in LMICs is associated with adverse child neurodevelopmental outcomes, although casual inferences cannot be drawn in the absence of well conducted prospective studies. Statistically significant correlations were described between parental alcohol misuse and child emotional and behavioural difficulties, cognitive delay, and risky behaviours. In future, prospective cohort studies are recommended, with adjustment for confounders.

**Supplementary Information:**

The online version contains supplementary material available at 10.1007/s10578-020-01112-3.

## Introduction

Experiences in childhood have been shown to have a significant impact both on concurrent health and development in later life [1–4]. This might be through exposure to social and environmental factors directly leading to the development of particular diseases, or more indirectly, with childhood experiences shaping attitudes and future health behaviours.

The growing evidence, predominantly from high-income countries, of the life-long impact of early adversity has led to the association of early adversity with adult disease outcomes such as poor self-rated health, diabetes, obesity, heart and lung disease, stroke, and cancer [5–7]. This association has even been extended to an increased risk of premature death [8, 9]. One possible mechanism for this association is through adversity encouraging health-harming behaviours and reduced self-efficacy as adults [10]. There is also a notable association between early adversity and various risk behaviours and mental health problems [11, 12] including adult alcoholism [13], depression [14], and suicidality [15]. However, while many of these aforementioned consequences become apparent later in adulthood, in some cases the impact of negative experiences on children can be observed in early life [16, 17].

Household substance abuse has been frequently cited as one of a number of adverse childhood experiences (ACEs), alongside mental illness, conflict, neglect, or abuse, which have been particularly associated with harmful child neurodevelopmental and behavioural outcomes [18]. In particular, a growing number of studies, primarily from high income countries, have documented the negative consequences of childhood exposure to a household member who misuses alcohol. Harmful or problem drinking by parents, caregivers, or others in the household can disrupt family relationships, place children under chronic mental, physical and physiological stress, and lead to injury, abuse and neglect. Associated consequences in terms of child health and development potential include developmental delays, cognitive impacts, behaviour problems in younger children, and a range of problem behaviours in adolescence such as absence from school, substance abuse, and teenage pregnancy. Studies in high-income countries have reported adverse neurodevelopmental outcomes regarding children’s cognitive and academic performance [19], adolescent alcohol and illicit drug use [3], and externalisation behaviour difficulties [20, 21]. Some studies suggest a dose-dependent relationship for adverse experiences whereby an accumulation of adverse exposures, including parental alcohol abuse, can lead to a higher risk of negative outcomes, including poorer health and mental health and later substance abuse by the child [1, 22, 23].

Since household alcohol consumption patterns are dependent on societal and economic factors affected by the culture and the income status of a country, it is not necessarily appropriate to extrapolate evidence gathered from high income settings to children from low- and middle-income countries (LMICs). Family units tend to be more cohesive in LMICs than in high-income countries, with intergenerational coresidence [24] potentially increasing the number of caregivers in the home who could engage in alcohol misuse around a young person [25]. The presence of multiple caregivers could also be a protective factor. Thus far there has not been a systematic review of the impact of household alcohol misuse on children from LMICs. This paper aims to synthesise the results of studies from subjects living in low-and middle-income countries, in the hope that findings can inform directions for future research and potentially inform policy recommendations.

In particular, there is evidence to indicate that the impact of exposure on children under 13 years compared to adolescents may differ. Brain development undergoes rapid advancement in the early years; for example it quadruples in weight, and acquires 90% of adult volume, before the age of six [26]. Early childhood stressors lead to increased activation of the hypothalamic–pituitary–adrenal axis and glucocorticoid release, causing changes in glucocorticoid-sensitive areas of the brain such as the hippocampus, amygdala and prefrontal cortex [27, 28]. In a rat model, early exposure to stressed and abusive caretakers led to persistent changes in methylation of brain-derived neurotrophic factor DNA and subsequent gene expression in the prefrontal cortex [29]. In a study of MRI scans of children exposed to early life stress, these children had smaller hippocampal and amygdala volumes than controls [30]. In later childhood, grey matter volume relative to white matter peaks, and later decreases during adolescence [31]. Adolescent brain development occurs asymmetrically with the limbic system and reward system developing faster than the prefrontal cortex, believed to explain why adolescents may tend towards risk-taking behaviour [32]. Furthermore, behavioural and neurodevelopmental changes in adolescence are affected by the onset of puberty and concurrent hormonal changes leading to changes in neuronal development and cognitive function [33, 34]. The neurodevelopmental influence of exposures will therefore differ according to the age at which they are experienced, the number of times they have been experienced, and potentially the age at which a young person receives medical care. Therefore an independent examination of the evidence for the impact of household alcohol consumption on children, relative to adolescents, is useful. This particular review article focuses on childhood developmental outcomes, specifically children aged 0–12-years-old, alongside a parallel review focusing on adolescent behavioural outcomes [35].

1. To systematically identify studies of the impact of excess alcohol consumption among household adults on child developmental health outcomes (neurodevelopmental, cognitive and behavioural) in low- and middle-income countries and to evaluate the quality of the research
2. To explore whether the nature of alcohol use and misuse differs by individual family members (father, mother or other family member) in its impact on child health outcomes (including neurodevelopment, cognitive and behavioural impact) in low- and middle-income countries

## Methods

A protocol for this review was published on the PROSPERO register in June 2017, registration number CRD42017070209.

### Eligibility Criteria

Studies were included in this review if they met the following criteria:

- Participants: children and young people aged 0–12 years
- Exposure: adult household member engaging in alcohol misuse
- Setting: low- and middle-income countries as defined by the World Bank [36]
- Outcome: Outcome measure of adverse child behavioural and neurodevelopmental impacts (excludes outcomes directly related to alcohol exposure such as children’s own drinking behaviour as a result of adult alcohol exposure or in utero alcohol exposure)
- Language: Studies published in English, or with translation available
- Year: Published from 1990 or later

An initial pilot search had revealed that a number of varying terms were in use in the literature to describe disorders related to alcohol consumption, including terms ‘problem drinking’, ‘hazardous alcohol use’ ‘alcoholism’, ‘drunkenness’, ‘alcohol abuse’, ‘alcohol addiction’ amongst others. Between studies, there were also inconsistencies in defining these terms. For example, some authors used these terms strictly under the remit of validated alcohol screening questionnaires, whereas others defined exposure to alcohol more loosely and casually. Therefore, ‘alcohol misuse’ i.e. harmful use (ICD-11 code F10.1), is used throughout this paper in order to be consistent with the World Health Organisation, International Classification of Mental Disorders, 11th Revision (ICD-11) [37]. Studies with participants aged 0–12 years old were considered eligible; studies which only included a small minority of participants within this range were excluded. Studies which focused on antenatal, rather than household, alcohol exposure, were excluded due to the confounding influence of intrauterine toxic exposure to alcohol. ACE studies which included parental alcohol as one of a cumulative list of adverse childhood experiences without detailing the individual effect of alcohol misuse were excluded.

### Information Sources

Five electronic databases were searched from 1990 to April of 2020: Medline, EMBASE, OVID Global Health, Cochrane Library and PsychInfo. An original June 2017 search was updated in June 2018 and in April 2020.

### Search Strategy

The search strategy was structured as follows: “alcohol use” AND “household” AND “young person” AND “neurodevelopmental outcome(s)” with associated synonyms. The full search strings used for MEDLINE are available in Supplementary File 1*.* In order to ensure that no potentially relevant studies would be missed, the search term included alcohol ‘use’ to be deliberately broad and inclusive.

The returned results were then secondarily sorted using the Cochrane LMIC filter to screen for studies set in low and middle income countries (LMICs) [38]. This filter has been extensively used in systematic reviews of similar nature [39–42]. During targeted abstract and full text review, further screening made use of the up to date World Bank list of LMICs [36].

### Study Selection and Data Extraction

After the studies were downloaded, and the Cochrane LMIC filter had been applied, each title and abstract was reviewed by one reviewer and uncertainties checked by a second against the inclusion criteria. Shortlisted full-text articles were subsequently checked by two separate reviewers. This led to the final list of studies to be included.

A standardised pre-piloted extraction form was developed, tested on 10 articles and revised iteratively as needed. Extracted information included:

1. Study characteristics: setting, study design, method of data-analysis;
2. Participants: study population, number of participants in each group, patient characteristics
3. Child or adolescent health outcome (as reflected in primary outcome)
4. Household adult alcohol exposure or definition (as reflected in secondary outcome)
5. Household location, income, food insecurity, asset index and family factors and other factors (if available)

Each study type was classified e.g. cohort, cross-sectional study, according to a standard definition [43]. Each study was also classified as relevant to adolescents, children, or both.

### Results Synthesis

Because of the heterogeneity of both exposures and outcomes, the evidence reviewed is presented as a narrative report with results broadly categorized by outcome within the following categories:

- Child behavioural problem/disorder
- Child cognitive delay/disorder
- Risky behaviour
- Other

### Quality Assurance

The National Heart, Lung and Blood Institute (NHLBI) Quality Assessment Tool for Observational Cohort and Cross-Sectional Studies was utilised to assess the quality of the included studies, or the equivalent tool for Case–Control studies if applicable [44]. The former tool asks 14 questions with answers of ‘Yes’, ‘No’ or ‘Other’, such as ‘*Was the exposure(s) assessed more than once over time?’* Two reviewers independently screened each study, with additional arbitration where required to reach an overall score. A maximum score of 14 (12 for Case–Control studies) was available for each paper, and a minimum score of 0, with higher scores indicating a high-quality paper relevant to our objectives. The scores were then used to produce an overall rating of ‘Good’, ‘Fair’ or ‘Poor’ relevant to the review, with studies rated as ‘Poor’ to be excluded from inclusion in the main results section.

## Results

**Fig. 1:**
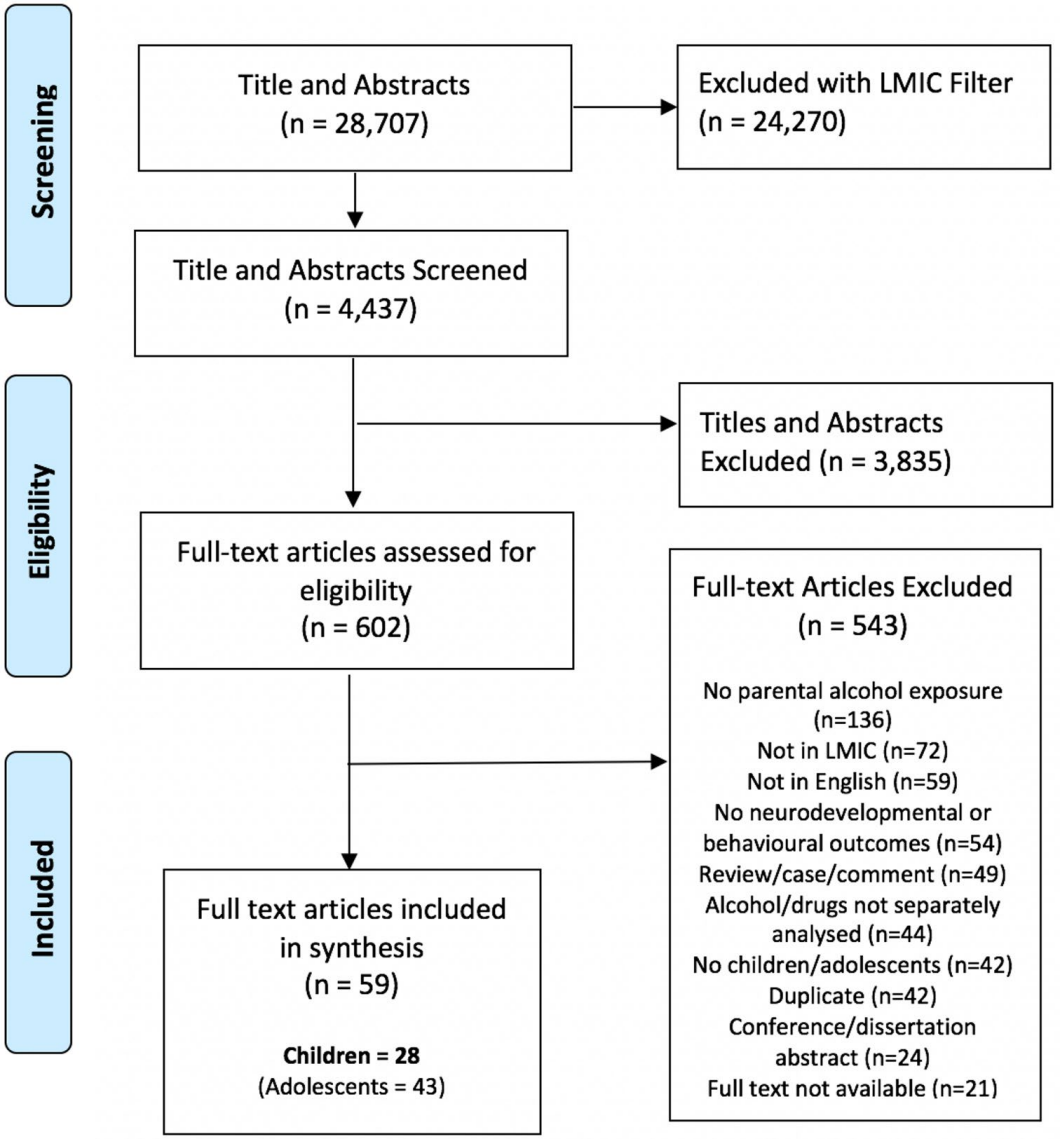
Study selection process (Color figure online)

In total, 28,707 titles and abstracts were downloaded from the chosen databases. After addition of further filtering (namely the Cochrane LMIC filter [38]), 4,437 results were screened by title and abstract. Following this, 602 papers were selected for full text review. The process of study selection is illustrated in Fig. 1*.*

### Study and Participant Characteristics

In total, 59 studies were identified and relevant to individuals aged 0–18 years. Of these, 28 studies were focused on children 0–12 years old, and were included in this review. The adolescent studies are summarised elsewhere [35].

Of these 28 studies relevant to outcomes in children, 18 were cross-sectional, five were case–control and five cohort studies. The studies came from 11 different low- and middle-income countries, with the most frequently studied country being India (8 studies); see Table 1. Across these studies, there were 42,599 children/participants, with the age range across the included studies varying from 1.5 years to 20 years but predominantly focusing from 0–12 years (Table 2).

**Table 1:** Countries of origin of included studies

Country	Number of studies
India	8 studies [45, 47, 50, 51, 56, 59, 71, 76]
South Africa	6 studies [55, 58, 60, 61, 77, 79]
Thailand	3 studies [49, 54, 62]
Turkey	2 studies [48, 75]
Ukraine	2 studies [46, 63]
Brazil	2 studies [52, 72]
Russian Federation	1 study [57]
Rwanda	1 study [74]
Belarus	1 study [53]
China	1 study [73]
Malaysia	1 study [78]

**Table 2:** characteristics of the included studies

Author	Country	Study type	Sample size	Population	Age range	Household alcohol exposure	Household location	NHLBI Quality score	NHLBI Overall Assessment
Ahmed et al. [78]	Malaysia	Cross-sectional study	3509	Primary school children	10–12 years	Father /Mother	Mixed	10	Good
Bele et al. [45]	India	Cross-sectional study	370	Children in a slum area	5–10 years	Father	Urban slums	6	Fair
Betancourt et al. [74]	Rwanda	Cross-sectional study (as part of larger Case–control study)	680	Children /adolescent with/without HIV	10–17 years	Family	Rural	6	Fair
Burlaka et al. [63]	Ukraine	Cross-sectional study	251	Children participating in the Ukrainian Child and Family Study	9–16 years	Mother	Mixed	9	Good
Chander et al. [58]	South Africa	Cross-sectional study	980	Interview of pre-school children and caregivers in the Asenze Study	4–6 years	Caregiver	Peri-urban	9	Fair
da Rocha et al. [72]	Brazil	Case–control study	146	Those with an IQ less than 70 referred to a special education institution	7–19	Relative	Urban	6	Fair
Demirci et al. [75]	Turkey	Cross-sectional study	1969	Children and youth seeking treatment for substance abuse	11–20 years	Parents/relatives	Urban	8	Fair
Drabick et al. [46]	Ukraine	Cross-sectional study	600	Children evacuated from around Chernobyl and controls	10–12 years	Father	Urban	7	Fair
Jardin et al. [55]	South Africa	Cross-sectional study	742	Children orphaned by AIDs-related illnesses or other illnesses. Non-orphaned children	7–11 years	Caregiver	Urban	10	Good
Jogdand et al. [47]	India	Cross-sectional study	600	Children in an urban slum area	6–18 years	Parents and care-giver(s)	Urban slums	7	Fair
Kheokao et al. [54]	Thailand	Cross-sectional study	5184	School students	Grades 4^th^-12th	Family	Not reported	8	Fair
Kilic and Şener [48]	Turkey	Cohort study	92	Diagnosed with ADHD	6–11 years	Father	Mixed	7	Fair
Krishnakumar et al. [76]	India	Cohort study	29	Children with history of deliberate self-harm who were referred to clinic	6–12 years	Parents	Not reported	7	Fair
Mansharamani et al. [59]	India	Cross-sectional study	100	Outpatients and inpatients at a psychiatric ward of a tertiary facility	4–14	Parents	Mixed	6	Fair
Meyer et al. [49]	Thailand	Cross-sectional study	23	Children/adolescents in refugee camp	9–17 years	Parents /caregiver (s)	Refugee camp	6	Fair
Narang et al. [50]	India	Case–control study	200	Son(s) only	4–12 years	Father	Not reported	6	Fair
Nothling et al. [61]	South Africa	Cohort study	70	Children with HIV	42 months	Mother (biological or as primary caregiver)	Urban	10	Good
Pajarn et al. [62]	Thailand	Case–control study	110	Children attending an outpatient department	3–4 years	Parents	Not reported	7	Fair
Pillay and van der Veen [79]	South Africa	Cohort study	100	Children admitted to an inpatient psychiatric unit	4–17 years	Parents	Urban	6	Fair
Rahi et al. [51]	India	Cross-sectional study	620	Children from an urban slum area	4–14 years	Father	Urban slums	7	Fair
Raman et al. [56]	India	Case- control study	32	Children of men with and without alcohol dependence	5–9 years	Father	Not reported	6	Fair
Rochat et al. [60]	South Africa	Cohort study	1536	Children from the Siyakhula cohort	7–11	Mother/caregiver	Rural	10	Good
Ruchkin et al. [57]	Russia	Cross-sectional study	692	Children enrolled in government childcare centres	1.5–3.5 years	Mother	Urban	9	Good
Shenoy et al. [71]	India	Cross-sectional study	129	Children in Bangalore	5–8 years	Father	Urban	7	Fair
Wood et al. [77]	South Africa	Cross-sectional study	20	Children and adolescents convicted of sex offences	7–15 years	Parents/household	Not specified	6	Fair
Xing et al. [73]	China	Cross-sectional study	12,470	High school students	11–19 years	Family	Mixed	8	Fair
Yang and Kramer [53]	Belarus	Cross-sectional study	11,305	Belarusian children participating in a trial regarding breastfeeding intervention (PROBIT)	6.5 years	Father	Mixed	10	Good
Zanoti-Jeronymo et al. [52]	Brazil	Case–control study	40	Children of fathers admitted to psychiatric unit with alcohol abuse	10–12 years	Father	Mixed	7	Fair

### Quality Appraisal

The NHLBI quality scores of the included studies are listed in Table 2*.* All studies included in this review are rated as either Fair (n = 21) or Good (n = 7) (Table 2). The most common reason for studies to score poorly was due to failure to measure continuous data for an independent variable (i.e. alcohol consumption), and for not defining alcohol consumption or misuse by any validated tool.

Most studies included in this review did not, or insufficiently, report information related to household income/deprivation, assets index, food security, mental health and other related variables which may act as confounders or a mediating factor in childhood neurodevelopmental or behavioural outcomes when exposed to household alcohol misuse.

### Household Alcohol Exposure

The exact nature of household alcohol exposure varied between studies. Across the 28 studies, 15 studies examined the impact of generic parental/caregiver/familial alcohol consumption on neurodevelopmental and behavioural outcomes in the child. Nine studies examined exposure to paternal alcohol consumption, and four studies looked at the effect of maternal alcohol consumption (Table 2).

### Neurodevelopmental and Behavioural Outcomes

The most commonly investigated outcome was child behavioural problem or disorder, followed by child cognitive delay or disorder, and risk behaviour. The following sections examine the evidence for each of these outcomes in turn.

#### Child Behavioural Problem or Disorder

Neurodevelopmental aspects related to child behavioural problems/disorder was most frequently reported outcome. The outcome was measured in 19 of 28 included studies. Overall, 10 studies [45–54] described an overall significant correlation and/or association between alcohol exposure in household and a higher prevalence and/or severity of childhood behavioural problems/disorders. Six papers showed mixed results [55–60], whereas three papers [61–63] did not demonstrate any significant correlation and/or association.

A number of studies used validated questionnaires to assess childhood behavioural problems which included the Strengths and Difficulties Questionnaire [45, 53, 55, 58, 62], Childhood Behaviour Checklist (CBCL) [47, 56, 57, 60, 61], Childhood Psychopathology Measurement Schedule (CPMS) [50, 51, 59], the Rutter A2 Scale of Behaviour [52], and Youth Self-Report [63], whereas three studies [46, 48, 59] included development disorders, namely oppositional defiant disorder (ODD), Attention Deficit/Hyperactivity Disorder (ADHD) and Conduct Disorder (CD) as neurodevelopment outcomes (Supplementary Table S1).

Five studies explored child behaviour outcomes using the Strengths and Difficulties Questionnaire (SDQ) [45, 53, 55, 58, 62]. The SDQ is used to assess emotional and behavioural problems among children across a number of subscales and can be used to screen for and monitor psychological disorders [64, 65]. It produces sub-scale scores for internalising and externalising behaviours, and the sum of these produces the total difficulties score. Overall, the five studies included 14,108 children with an age range of 3–11 years old, and showed an association between household member alcohol misuse and greater difficulties. Three studies [55, 58, 62] examined care-giver/parental alcohol misuse, and two examined father’s use of alcohol [45, 53]. Two cross-sectional studies in South Africa [55, 58] examining caregiver/parental alcohol exposure and showed an association with high total SDQ scores. In India, Bele et al*.* (2012) found having an alcoholic father was a significant predictor of scores showing an emotional or behavioural disorder [45]. In a Belarusian cross-section study of 11,305 children, authors found increased total difficulty SDQ scores in children whose fathers had heavy or moderate drinking habits (difference in score 0.9, 95% Confidence Interval (CI) 0.5–1.4 and 0.5, 95% CI 0.1–0.8 respectively) [53]. However, when stratifying scores, they found an association with increased scores for externalising behaviours (hyperactivity and conduct problems) but not for internalising behaviours (emotion and peer problems) after adjusting for confounders [53]. In contrast, in Thailand, Pajarn et al*.* (2012) did not demonstrate an association between parental alcohol misuse and SDQ scores [62].

The Child Behaviour Checklist (CBCL) is an instrument designed to assess behavioural and emotional disorders among children aged 4–16 years, with good reliability and validity in a variety of cultural and language settings [66–69]. It has eight domains, namely: emotionally reactive, anxious/depressed, somatic symptoms, withdrawn behaviour, sleep problems, common/non-specific problems, attention problems and aggressive behaviour. The first four domains are categorised as internalising behaviour problems and the latter two as externalising behaviour problems. Five studies [47, 56, 57, 60, 61], reported child behavioural outcomes using CBCL with one study demonstrating a clear association [47]. These studies included children from South Africa (n = 3), India, and Russia (Table 3). Jogdand et al*.* (2014) in India showed a clear association between alcoholism in the parent and overall higher odds of behavioural problems (Odds Ratio (OR) 1.56 (CI 1.12–2.17) p < 0.05) [47]. In another Indian study, Raman et al*.* (2010) showed higher scores in externalisation behaviour (p < 0.01), but no significant difference in internalising behaviour [56]. In South Africa, Rochat et al*.* (2019), children of mothers with hazardous drinking had higher mean scores for psychological problems (mean 45.0 if no hazardous drinking, 48.9 for hazardous drinking, p = 0.029) [60]. They also found a significant association in univariate logistic regression between any alcohol use by mothers and children meeting referral thresholds for internalising/externalising problems, but this was no longer significant in multivariate analyses. In a second South African study, Nothling et al*.* (2013)*,* found that maternal alcohol abuse did not have significant explanatory power for child behaviour problems in various models [61]. Ruchkin et al*.* (2008) in Russian children found children’s aggressive and destructive behaviour to be significantly and positively (albeit weakly) correlated with alcohol amount, but not with alcohol frequency [57]. In an Ukrainian study, Burlaka et al*.* (2017) used the Youth Self-Report questionnaire (which is a parallel form to the CBCL) specifically looking at maternal alcohol use, and found no statistically significant correlation with child internalisation problems [63].

**Table 3:** Child behaviour problem or disorder

Author	Context of study	Neurodevelopmental /behavioural outcomes	Unadjusted results	Adjusted results	Confounders adjusted?
Bele et al. [45]	The authors aimed to examine the prevalence of, and risk factors for, emotional and behavioural disorders in children in urban slums	Overall emotional or behavioural disorders measured by SDQ (which includes conduct problems, emotional symptoms, pro-social behaviours, hyperactivity, peer problems, and total difficulties)	OR 11.65 (p = 0.000)	–	No adjustment
Burlaka et al. [63]	The authors examined the association between internalisation behaviours in children and household incomes, maternal education, age, depression, alcohol use, and parenting techniques	Internalisation of behaviours by face-to-face interviews using Youth Self-Report questionnaire	Maternal alcohol use and child internalisation of behaviours, p = 0.326	Maternal alcohol use and child internalisation behaviours were not significantly associated in multiple regression analysis; B = -0.12, p = 0.326	Child and mother sociodemographics, maternal depression, maternal alcohol use, parenting practices
Chander et al. [58]	Examined the association between caregiver’s experience of Intimate Partner Violence (IPV) and behaviour in children within the age range of 4–6	Internalisation, externalisation and total behavioural problems using the Strength and Difficulties questionnaire (SDQ)	In household with IPV exposure, association between caregiver binge drinking and total SDQ score (OR 2.136, 95% CI 1.261–3.618, p = 0.0047)	–	No adjustment
Drabick et al. [46]	Examined potential external validators for the differences in oppositional defiant disorder (ODD) and Attention Deficit/Hyperactivity Disorder (ADHD). The authors also examined whether co-occurrence of ODD and ADHD results in any additive or synergistic pattern of impairment		Fathers of children with ADHD were more likely to abuse alcohol than fathers of children with ODD ± ADHD (p < 0.05) in teacher defined subgroups	–	No adjustment
Jardin et al. [55]	To examine the effect of caregiver alcohol-use problems on the impact of AIDS-affect orphaned children compared to non-AIDS-affected children (orphaned or non-orphaned)	Overall emotional and behavioural disorders measured by SDQ	-	Correlation between teacher reported SDQ and caregiver alcohol use problem B = 0.075, p < 0.05. Others reported sources of SDQ were non-significant including child self-report and caregiver-reported SDQ	AIDS-orphans vs. non-AIDS orphaned children
Jogdand et al. [47]	To examine the association between family factors and behaviour problems in children	Externalisation and internalisation behaviours	Association between alcoholism in parents and prevalence of behaviour problems OR 1.56 (CI 1.12–2.17) p < 0.05	–	No adjustment
Kheokao et al. [54]	The study examined correlations between drinking intention, alcohol use, school, family, media factors	Absenteeism	There was a significant correlation between family drinking and absenteeism (p < 0.05)	–	No adjustment
Kilic and Şener. [48]	The authors compared developmental, sociodemographic and behavioural/emotional aspects of children with ADHD alone or those with accompanying ODD and/or CD	ADHD, ODD, and CD	Paternal alcohol abuse rates were significantly higher in ADHD subjects with ODD and/or CD comorbidities (p < 0.05), compared to ADHD alone	–	No adjustment
Mansharamani et al. [59]	Assessed psychiatric morbidity in children of alcoholics and compared with children of non-alcoholic parents	Total Score of Children Psychopathology Measurement Schedule: also assessed sub-factors e.g. depression	Significant difference in ‘Total Score of Children Psychopathology Measurement Schedule’: mean 6.10 in children of alcoholics, 3.12 non-alcoholics, p = 0.0001. No difference in psychotic symptoms, no difference in special symptoms, no difference in conduct disorder	–	No adjustment
Meyer et al. [49]	The authors examined chronic stressors for children in a refugee camp	Qualitative study using thematic analysis of interviews; behavioural effects included not going to school, going against parents, hiding, and emotionally: feeling afraid, shy, crying	–	–	–
Narang et al. [50]	Investigate the psychopathology and temperamental characteristics of children with alcoholic fathers	Psychopathological measurements (Low intelligence with emotional problems, conduct disorder, anxiety, depression, psychotic symptoms, special symptoms, physical illness with emotional problems and somatization); Temperament measure (approach withdrawal, adaptability, threshold of responsiveness, mood, persistence, activity level, intensity, distractibility, rhythmicity)	Conduct disorder (mean score 5.85, p < 0.01); Anxiety (mean score 3.10, p < 0.01), somatization (mean score 1.83, p < 0.01), Mood (mean score 3.16, p < 0.01), persistence (mean score 2.93, p < 0.01), Rhythmicity (mean score 2.89, p < 0.01) *mean score lower in children of alcoholic fathers than non-alcoholic fathers	–	No adjustment
Nothling et al. [61]	To investigate, in mother–child dyads infected with HIV, the impact of maternal postpartum trauma exposure and PTSD and their association with child behaviour	8 behavioural domains of CBCL	Maternal alcohol abuse (using AUDIT) in children with mothers who abuse alcohol vs children of mothers who did not abuse alcohol: t-test = 0.49; p = 0.628	Total problems (B = -0.02 p = 0.894), Internalisation problems (B = -0.13, p = 0.328), Externalisation problems (B = 0.15, p = 0.270)	Depression, Traumatic life events, PTSD, functional disability
Pajarn et al. [62]	To determine the impact of parental drinking problem on emotion and behavioural problems in pre-school children	Emotional and behavioural problems using SDQ	Total abnormal behaviour (OR 1.07 CI 0.508–2.27, p = 1.0); Hyperactivity (OR 1.20 CI 0.40–3.4, p = 0.79); Emotional problem (OR 1.17, CI 0.07–19.3, p = 1.0) Conduct problems (OR 0.85, CI 0.34–2.1, p = 0.81); Peer problem (OR 0.14, CI 0.016–1.2, p = 0.06); Pro-social behaviors (OR 0.40, CI 0.14–1.17, p = 0.12)	–	No adjustment
Rahi et al. [51]	Explore the role of demographic, developmental and social factors on the effect of psychopathology in children in an urban slum area in India	Psychopathological disorders using Childhood Psychopathology Measurement Schedule (CPMS)	Psychopathological disorder (CPMS score > 10) was significantly higher in children of alcohol abusing fathers compared to non-alcohol abusing fathers (p < 0.05) (prevalence 20.2% and 13.6% respectively)	–	No adjustment
Raman et al. [56]	Examined a wide range of dysfunctions in children of fathers with alcohol dependency	General neurodevelopment, Child behaviour checklist (CBCL), IQ scale, Trail making test (TMT), Malin’s Intelligence Scale for Indian Children	In children of alcohol-dependent fathers, mean score was higher in Internalisation problems (NS), Externalisation problems (p < 0.01), and Neurodevelopment examination (p < 0.001). It was lower in IQ scale verbal (p < 0.001), IQ scale performance (p < 0.01), and full IQ scale (p < 0.05)	–	No adjustment
Rochat et al. [60]	Explored the association between alcohol use, hazardous drinking and child behaviour and cognition	Psychological problems including CBCL total score, above threshold for internalising problems and externalising problems	Children of mothers with hazardous drinking had higher mean scores for psychological problems (mean 45.0 for no hazardous drinking, 48.9 for hazardous drinking, p = 0.029). There was a significant difference in percentage of children above threshold for referral for investigation of internalising problems and externalising problems in those with any alcohol use (univariate analyses)	There was no significant difference in percentage of children above threshold for referral for investigation of internalising problems (OR 1.06, p = 0.851) and externalising problems (OR 1.58, p = 0.107) in those with any alcohol use vs none, after adjusting in multivariate logistic regression	Mother/caregiver age and education, mother being primary caregiver, mother’s relationship status, HIV status, employment, household food insecurity, ownership of a fridge, child age, sex
Ruchkin et al. [57]	Assessed association between multiple biological and psychological risk factors in mothers and behavioural outcomes in children	Emotional and behavioural problems	Pearson correlation coefficient between a number of variables Non-significant: - Aggressive behaviour and alcohol frequency, .04, NS - Destructive behaviour and alcohol frequency, .05, NS - Withdrawn behaviour and alcohol frequency -.05, NS; and alcohol amount, -.01 NS - Anxious/depressed behaviour and alcohol frequency -.02, NS; and alcohol amount -.01, NS Significant: - Aggressive behaviour and alcohol amount, .11, p < 0.01 - Destructive behaviour and alcohol amount .09 p < 0.05 Correlation coefficient between current maternal alcohol consumption and: - Externalising problems: .09, NS - Internalising problems: -.09, NS	Structured equation modelling found an acceptable fit for internalising and externalising problems; however, current maternal alcohol consumption was eliminated from the model as it did not indicate significant pathways to child behaviours	Current maternal and family dysfunction, prenatal alcohol consumption
Yang and Kramer [53]	The authors examined the associations between paternal alcohol consumption and behavioural problems in Belarusian children, effect on family transition and their cognitive ability	Intelligence and behavioural outcomes using SDQ	–	Paternal weekly moderate or heavy drinking was associated with children’s lower full-scale IQ scores: 1.6 (95% CI -2.5, -0.9) and 2.5 (95% CI -3.4, -1.6) respectively, compared with those whose fathers were infrequent or light drinkers Frequent paternal drinking (both amongst moderate and heavy drinkers) was associated with increased scores in total difficulties and externalising behaviours. However, it was not associated with internalising behaviours following confounders adjustment	Gestation age at birth, birthweight, sex, maternal and paternal age at the birth of child, maternal alcohol and smoking consumption during antenatal period, breastfeeding, number of older children in the household (proxy for birth order), both parents’ education, occupation, paternal smoking and maternal drinking
Zanoti-Jeronymo et al. [52]	This study measured participants’ behavioural problems, self-concept, cognitive level and academic performance under the environment of an alcoholic father	Self-concept, children’s behaviour (health problems and behaviours), academic performance by psychometric instrument, Evolution and emotional status by human figure drawing test	Overall self-concept score, academic performance and emotional and behavioural aspects better in children of non-alcoholic fathers (p = 0.00003, 0.0011, 0.00016 respectively)	–	No adjustment

Abbreviation: NS = Not significant

Three studies used the Childhood Psychopathology Measurement Schedule (CPMS) to measure behavioural outcomes in Indian children exposed to household paternal alcohol use [50, 51, 59]. CPMS was originally developed in India by Malhotra et al*.* (1988), which is an adaptation of the CBCL for Indian children [70]. Narang et al*.* (1997) and Rahi et al*.* (2005) found significantly higher score in negative behaviours and lower scores in positive behaviours in those exposed to household paternal alcohol consumption *(*Table 3*)* [50, 51]. Mansharamani et al*.* (2018) described significantly higher mean total CPMS scores in children of alcoholics compared to non-alcoholics [59]. Adjustment for potential confounding factors did not take place in these studies.

In Brazil, Zanoti-Jeronymo et al*.*[52] used the Rutter A2 Scale of Behaviour in Children and found an overall higher score in emotional and behavioural problem aspects in children of alcoholic parents compared to non-alcoholic parents (Median 16.5 vs 8, p = 0.00016). In the only qualitative study included in this systematic review, Meyer et al. (2013) examined the effect of chronic stressors on refugee children in the Ban Mai Nai Soi camp in Thailand [49]. The authors reported parental drinking led to children ‘feeling upset’, ‘shy’, ‘depressed’, and ‘having difficulty concentrating’.

The presence of Attention Deficit Hyperactive disorder (ADHD), Oppositional Defiant Disorder (ODD), Conduct Disorder (CD), Obsessive Compulsive Disorder (OCD) were addressed by studies in Ukraine [46], Turkey [48] and India [59]. Drabick et al*.* (2004) found that fathers of children with ADHD more commonly abuse alcohol than fathers of children with ODD ± ADHD in teacher defined subgroups [46]. Conversely, Kiliç and Şener (2005) found that paternal alcohol abuse was more common in children with ADHD and ODD and/or CD compared to ADHD alone [48]. However, Mansharamani et al. (2018) found no difference in scoring for OCD and Conduct disorder between children of alcoholics and children of non-alcoholics [59] (Table 3).

#### Child Cognitive Delay or Disorder

Eight studies [52–54, 56, 59, 60, 71, 72] investigated cognitive delay/disorder related to intelligence and academic performance (Table 4). Overall, most studies found an association between paternal drinking and lower performance in key academic domains and/or intelligence. Importantly, there was some indication of a dose-dependent nature of paternal alcohol consumption and child’s IQ score [53].

**Table 4:** Child cognitive delay or disorder

Author	Context of study	Neurodevelopmental/behavioural outcomes	Unadjusted results	Adjusted results	Confounders adjusted?
da Rocha et al. [72]	The study reported MRI results of children whose IQ scored less than 70	IQ < 70 and associated structural brain lesions	Of this sample of children with an IQ < 70, 62% of children in the group with no structural lesions in the brain, and none (0%) of children where some structural lesion was found, had at least one relative with a history of alcoholism	–	No adjustment
Kheokao et al. [54]	The study examined correlations between drinking intention, alcohol use, school, family, media factors	Grade point average (GPA)	There was no significant correlation between family drinking and GPA (p > 0.05)	–	No adjustment
Mansharamani et al. [59]	Assessed psychiatric morbidity in children of alcoholics and compared with children of non-alcoholic parents	Relevant to child cognition: assessed low intelligence with behavioural problems, among other outcomes	There was no difference in ‘low intelligence with behavioural problems’ as per Childhood Psychopathology Measurement Scale: 0.70 for children of alcoholics, 0.82 for children of non-alcoholics, p = 0.66	–	No adjustment
Raman et al. [56]	Examined a wide range of dysfunctions in children of fathers with alcohol dependency	General neurodevelopment Child behaviour checklist (CBCL), IQ scale, Trail making test (TMT), Malin’s Intelligence Scale for Indian Children	In children of alcohol-dependent fathers, mean score was higher in Internalisation problems ( NS), Externalisation problems (p < 0.01), and Neurodevelopment examination (p < 0.001). It was lower in IQ scale verbal (p < 0.001), IQ scale performance (p < 0.01), and full IQ scale (p < 0.05)	–	No adjustment
Rochat et al. [60]	Explored the association between alcohol use, hazardous drinking and child behaviour and cognition	KABC Learning Scale: cognitive scores	Children of hazardous drinking mothers/caregivers were significantly more likely to have lower scores on the KABC Learning Scale: lower cognitive scores for learning (no hazardous drinking mean 14.3, vs hazardous drinking mean 12.8, p = 0.017) and riddles solving (no hazardous drinking mean 4.1, vs hazardous drinking mean 3.6, p = 0.045). However, no significant effect for sequential cognition, planning, or simultaneous	–	No adjustment
Shenoy et al. [71]	Prevalence of scholastic backwardness and psychological disturbance and associated psychosocial aspects in children five to eight years old	Scholastic backwardness reported as by teachers	–	Regular drinking in father was more common amongst scholastically backward children than scholastically superior children (41.94% and 23.88% respectively, p < 0.05)	Matched scholastically backward and superior children on age, gender, and school class level
Yang and Kramer [53]	The authors examined the associations between paternal alcohol consumption and behavioural problems in Belarusian children, effect on family transition and their cognitive ability	Intelligence	–	Paternal weekly moderate and heavy drinking was associated with lower full-scale IQ scores by 1.6 (95% CI -0.9—-2.5) and 2.5 (95% CI -1.6—-3.4) points, respectively, compared with those whose fathers were infrequent or light drinkers	Gestation age at birth, birthweight, sex, maternal and paternal age at the birth of child, maternal alcohol and smoking consumption during antenatal period, breastfeeding, number of older children in the household (proxy for birth order), both parents’ education, occupation, paternal smoking and maternal drinking
Zanoti-Jeronymo et al. [52]	This study measured participants’ behavioural problems, self-concept, cognitive level and academic performance under the environment of an alcoholic father	Self-concept, children’s behaviour (health problems and behaviours), academic performance by psychometric instrument, Evolution and emotional status by human figure drawing test	Overall self-concept score, academic performance and emotional and behavioural aspects better in children of non-alcoholic fathers (p = 0.00003, 0.0011, 0.00016 respectively)	–	No adjustment

In a cross-section study in India, which categorised 129 children into either scholastically ‘backward’ or ‘superior’, Shenoy et al*.* (1996) found that children who are ‘scholastically backward’ more commonly have fathers who drink regularly (41.94%), compared to children who were identified as ‘scholastically superior’ (23.88%, p < 0.05) [71]. Similarly in Brazil, authors found children of alcoholics had a overall score in an academic performance test (which included reading and arithmetic), compared to children of non-alcoholic fathers [52]. Raman et al*.* (2010) found, using Malin's Intelligence Scale for Indian children, mean score (93.28) for the verbal section in children of alcohol-dependent fathers was significantly lower than children of non-dependent fathers (107.1, p < 0.001) [56]. Similar results are found in performance (Mean score 95.7 vs 108.2 respectively, p < 0.01) and full-scale IQ test (Mean score 96.14 vs 105.1 respectively, p < 0.05). The largest study addressing cognitive delay from household alcohol exposure came from a Belarusian cross-sectional study in 11,305 children aged 6.5 years-old using Wechsler Abbreviated Scales of Intelligence (WASI) [53]. It showed children whose fathers were at least weekly moderate and heavy drinkers had lower IQ scores by 1.6 points (95% CI -2.5—-0.9) and 2.5 points (95% CI -3.4—-1.6), respectively, compared with fathers who were light or infrequent drinkers. Importantly the authors of this study controlled for confounders (including maternal alcohol/smoking, parent’s education, occupation, gestational age, and birth weight) (Table 4). In one MRI study sampling children with IQs < 70, da Rocha et al*.* (2006) found that 62% of children with no structural brain lesion had alcoholic relatives, compared to none of the children with structural brain lesions; however there was no adjustment for confounders [72].

Among studies with mixed or negative results, Rochat et al. (2019) found that children of mothers who engaged in “hazardous drinking” (Defined as scoring ≥ 8 on an AUDIT scale) were significantly more likely to have lower scores for learning (p = 0.017) and riddle solving (p = 0.045) using the Kaufman Assessment Battery for Children; however there was no significant effect on sequential, planning or simultaneous cognition [60]. Two other studies also found no significant impact of familial alcohol use on cognitive measures, one regarding the prevalence of ‘low intelligence with behavioural problem’ [59] and one on grade point average (GPA) [54].

#### Childhood risky behaviour

Six studies [49, 73–77] included information on childhood risky behaviour in relation to household alcohol consumption (Table 5). These risky behaviours included suicide attempts, self-harm, substance abuse/misuse and anti-social behaviour. It is important to note that since self-harm is not common in very young children, these studies primarily included subjects whose ages extended into adolescence, though they did include children less than 13-years-old (age ranges in the four main relevant studies were 10–17 [74], 11–20 [75], 9–17 [49], 11–19 [73]).

**Table 5:** Child risky behaviour

Author	Context of study	Neurodevelopmental/behavioural outcomes	Unadjusted results	Adjusted results	Confounders adjusted?
Betancourt et al. [74]	The authors discussed the challenges and success of performing mental health research among vulnerable children and adolescents in Rawanda	Non-fatal suicidal behaviour	Out of 20 cases reporting current suicidality and non-fatal suicidal behaviour, n = 4 (20%) reported alcohol abuse in family	–	No adjustment
Demirci et al. [75]	Sociodemographic characteristics and substance use pattern in children seeking treatment	Substance abuse	–	Alcoholism in parents and cannabis use (AOR 0.933 CI 0.581–1.497, p = 0.774), solvent/inhalants use (AOR 1.030 CI 0.707–1.499, p = 0.878), Ecstasy use (AOR 1.030 CI 0.707–1.499, p = 0.878), 2 or more substance use (AOR 1.145, CI 0.721–1.819, p = 0.566)	Age at the onset of substance use (mean years), gender, age at the initiation of treatment, history of psychiatric disorders, substance use history, criminal history of parents, living together with parents and history of alcohol and drug use in relatives (including parents)
Krishnakumar et al. [76]	Examined the nature of deliberate self-harm and associated factors	Deliberate self-harm (consumption of poisonous substance or attempted self-harm)	Parental alcoholism was found as a stress factor in n = 1 (3%) child	–	No adjustment
Meyer et al. [49]	The authors examined chronic stressors for children in a refugee camp	Qualitative evidence: children go against parents; children do not attend school	–	–	–
Wood et al. [77]	Descriptive study of psychological and sociological background of child sex offenders	Sex offences	Prevalence of alcohol abuse amongst 1 + family members was 75%; amongst both parents, 40%; amongst a cohabiting family member, 35%	–	No adjustment
Xing et al. [73]	Prevalence of and family factors associated with suicide attempts among high school students in China	Suicide attempts	Higher rates of suicide attempts in families with a family member having an alcohol abuse problem (4.4% compared to 2.4%, p < 0.001)	Social problem of families including gambling, alcohol abuse problem and violations of law were grouped into one model and then demographic and family factors were control. This showed OR 1.27 (1.17–1.38) and 1.25 (1.15–1.35) both p < 0.001 respectively	Model controlled for demographic and life-style characteristics of families

Studies in Turkey [75] and Rwanda [74] did not demonstrate an association between paternal/maternal drinking and risky behaviours amongst children *(*Table 5*)*. Specifically, in the former study [75], the authors found that parental alcoholism did not significantly increase the odds of cannabis use, solvent/inhalants use and/or ecstasy use in their children. Betancourt et al*.* (2016) in Rwanda found 20 cases with current suicidality among 680 children, of whom only 4 (20%) reported alcohol abuse in the family [74]. However, this was a methodology study and did not set out to test hypotheses directly relevant to this review (Table 5).

In China, in an unadjusted analysis, higher rates of suicide attempts were found amongst older children and adolescents (aged 11–19) in families where a family member had a problem with alcohol abuse (4.4% vs 2.4%, p < 0.001) [73]. In a separate analysis, the authors modelled social problems of family members (which included gambling, alcohol abuse, and violation of law) and found these to be associated with a higher risk of self-reported suicide attempts in a model controlling for family demographics (Adjusted OR 1.27, 95% CI 1.17–1.38, p < 0.001) and when also controlling for family lifestyle factors (OR1.25, 95% CI 1.15–1.35, p < 0.001). Finally, Wood et al*.* (2000) completed a small study (n = 20) of child and adolescent sex offenders, and found that the prevalence of alcohol abuse amongst one or more family members was 75% [77]. The two other studies included a qualitative study describing alcohol as a chronic stressor, and another study where parental alcoholism was a stress factor for one child out of 29 engaging in self harm [49, 76].

#### Other

Raman et al*.* (2010) explored cultural and intellectual activity ratings among children of alcohol dependent fathers compared to children without this exposure [56]. The authors found that the children of alcoholics had significantly lower independence and intellectual/cultural orientation scores compared to controls, but this was not the case for other types of personal development scores (e.g. active/recreational scores).

Zanoti-Jeronymo et al*.* (2005) examined children’s self-concept by comparing 40 children of alcohol and non-alcoholic fathers [52]. The authors demonstrated that in areas of self-concept, children of non-alcoholic fathers scored significantly higher in attributes of physical appearance, happiness and satisfaction, anxiety management, intellectual status and popularity, when compared to children of alcoholic fathers. Finally, regarding depression, Mansharamani et al*.* (2018) found that there were significantly greater mean CPMS scores for depression (p = 0.016), and anxiety (p = 0.02) in children of alcoholics compared to children of non-alcoholics [59]. However, in contrast, in a large study of 3,509 children in Malaysia, Ahmed et al*.* (2015) found that children exposed to parental alcohol abuse did not have higher odds of a high depression score (‘CES-DC’ score over 31) compared to those who were not (Adjusted OR 1.08 (95% CI 0.65 -1.80) [78]. Pillay and van der Veen (1997) compared characteristics of admissions to a child psychiatric facility and found there was no statistically significant association between being from a substance-abusing household (93% alcohol-implicated), and a diagnosis of depression, in a small sample in South Africa [79].

## Discussion

### What is Already Known on this Topic

This study contributes to the existing knowledge base on the impact of household alcohol exposure on neurodevelopmental outcomes in children, in particular children in LMICs. An important constituent of childhood growth is neurodevelopment, which includes the development of expressive and receptive language, including social communication; visual problem solving (nonverbal cognition); motor development; neurobehavioral development; and social-emotional development [80]. There are multiple factors including physiological and environmental, which can affect the normal trajectory of these developmental goals [81]. Home environment, and the people within, play a crucial role in this complex interaction [82–85]. Home environment provides nurturing ground for development, alongside adequate nutrition, education, protection from harm, abuse and neglect and good healthcare. However, it can also introduce experiences which may affect a child’s neurodevelopment and behaviour concurrently and which can endure into adulthood.

From studies based in high income countries, we know that exposure to parental alcohol abuse can cause profound cognitive, behavioural, emotional and other social problems in children [86–88]. Additionally, in terms of cognitive delay or dysfunction, a number of studies have shown that improvements in social supportive environments can result in subsequent improvements in cognitive scoring; low scores can reflect a non-supportive environment as much as any individual innate difficulty [89–91]. However, not all children with the same exposure to negative experiences, such as alcohol abuse within a household, may have an equal risk of developing a behavioural problem, with studies indicating a genetic influence on propensity towards certain behaviours [92, 93]. It is important to consider alcohol exposure in low- and middle-income countries as a separate entity, because the settings for studies conducted in high-income countries may be significantly different in terms of alcohol availability, drinking culture, family environment, family members, household locations (e.g. slums), poverty, and violence; these can all influence levels of alcohol exposure. The root cause of alcohol abuse in a household is also an important factor to consider. Numerous studies suggested that parental alcohol misuse might be a coping mechanism for dealing with family dysfunction and their own childhood exposure to life stress, such as child abuse [94, 95]. Thus, the underlying reason for negative childhood outcomes could be due to the presence of broader family dysfunction, with parental alcohol misuse acting more as a surrogate marker than a direct cause.

Family environment is a complex social environment with multiple adverse or protective issues at play. Adverse influences include loss or absence of a parent, job loss leading to leading to financial difficulties, poverty or low socio-economic status, neglect, household substance misuse including alcohol, and parental or family conflict and discord. By conceptualising family environment as a dynamic system, whereby change in behaviour in one element of the system impacts and causes compensatory changes by other family members, it can also be seen how children may adapt their behaviours, some of which may place them at risk, if a family member is absent or performing poorly due to alcohol abuse [96].

### What this Study Adds

To our knowledge, this is the first attempt to systematically review evidence regarding household alcohol exposure and its impact on neurodevelopmental outcomes in children in low- and middle-income countries. Despite heterogeneity in exposure and in settings, we report an overall association between household harmful use of alcohol and a number of negative neurodevelopmental outcomes in children, including behaviour problems, cognitive impairment and performance, fewer cultural/intellectual activities and low self-concept. However, evidence of an association between household alcohol misuse and risky behaviours was limited to studies which included a preponderance of adolescents.

Despite this observed association, household alcohol exposure has not been established as an independent risk factor for poor childhood neurodevelopmental outcomes. Only a handful of studies accounted for confounders such as parental depression, traumatic life events experienced by the household, financial problems, or childhood factors such as poor nutrition, premature birth, and intrauterine toxin exposure. Amongst the studies which adjusted for confounders, varied outcomes were investigated. In addition, the optimal method for drawing causal inferences regarding the role of household alcohol exposure should be through the use of well-designed prospective cohort studies; retrospective measures may be more vulnerable to bias [97]. In this review only three relatively small studies [48, 61, 76] were designed in this way. Future studies should establish temporal relationships between exposure and outcomes and adjust for confounders.

### Limitations in the Research Literature

#### Measurement of Alcohol Use

Loose definitions of 'alcohol abuse' or 'problem drinking' were used in many studies to compare outcomes with no quantification of what this might amount to. A range of tools have been reported- from standardised tools (e.g. AUDIT) to uncorroborated self-reports of alcohol abuse. The absence of uniform and quantifiable or clear functional measures of alcohol misuse precluded a comparison between studies. Consequently, it has not been possible to identify whether or not there is a level of household alcohol use or type of alcohol disorder or a dose response relationship that might constitute a risk to the behavior or development of their children. The nature of exposure to household alcohol was also poorly defined in the included studies. There was little indication of the extent to which children were directly exposed either to drinking or drunkenness; it was not clear whether alcohol use mostly occurred at home in the children's presence or outside the house. During assessment of full texts, it was noted that several of the studies matching our search terms were wholly or simultaneously addressing antenatal drinking. Since maternal antenatal drinking can have profound pathophysiological impact on the developing fetus, studies of maternal alcohol use should differentiate prenatal and postnatal exposure or clearly stratify results by timing of alcohol exposure.

### Research Implications

The heterogeneity in the results and the methodological challenges discovered necessitate that more studies be undertaken, with clearly defined neurodevelopmental outcomes and careful quantification of household alcohol use in order that more accurate conclusions can be drawn. In studies including a wide age range, the results should be stratified by age.

Further research also needs to be done to explore the mechanisms through which child neurodevelopmental outcomes might arise, and to elucidate the role of household alcohol use within these. Key covariates which could confound the relationship between household alcohol misuse and childhood behavioural or developmental disorders should be adjusted for. Furthermore, longitudinal studies could provide vital data on the temporal nature in which neurodevelopment is affected by household alcohol misuse, and thus could provide key information for appropriate interventional strategies.

### Limitations of this Review

Ultimately the strength of the conclusions of this review are limited by the methodological issues identified in the included papers. Due to the heterogeneous natures of the studies, we have carried out a narrative rather than a quantitative synthesis. This review only included studies which were published after 1990. Including only studies available in English meant that some potentially important studies could not be considered.

## Summary

This review shows that exposure to alcohol misuse by household members in the context of low- and middle-income countries is associated with adverse child neurodevelopmental outcomes, although causal inferences cannot be drawn in the absence of well conducted prospective cohort studies which address potential confounding. Nevertheless, the association was seen across a wide range of countries, in both urban and rural environments. Results were heterogeneous, which may be related to our observation that the types of alcohol misuse and the frequency and amounts of alcohol use were poorly quantified in many studies. Statistically significant correlations were demonstrated between paternal alcohol misuse and child problem behaviours, cognitive delay, and risky behaviours. In contrast, the association with maternal drinking was less well studied.

## Supplementary Information

Below is the link to the electronic supplementary material.

Supplementary file1 (docx 20 KB)
