# Supplementary material for: A Systematic Review of Household and Family Alcohol Use and Childhood Neurodevelopmental Outcomes in Low- and Middle-Income Countries
Source: Child Psychiatry Hum Dev. 2020 Dec 28;52(6):1194–217. doi: 10.1007/s10578-020-01112-3 (PMC8528783; doi:10.1007/s10578-020-01112-3)
Supplement: Supplementary file 1 — Supplementary file1 (docx 20 KB) [file 10578_2020_1112_MOESM1_ESM.docx]

**SUPPLEMENTARY FILE 1**

**Supplementary table 1: Questionnaire tools used by the included studies**

| **Author** | **Data collection tool** |
| --- | --- |
| Ahmed et al. (2015) | Questionnaire was adapted from a number of pre-validated instruments including the International Society for the Prevention of Child Abuse and Neglect (ISPCAN) Child Abuse Screening Tool Children’s Version, the Child Exposure to Domestic Violence Scale, Centre for Epidemiological Studies Depression Scale for Children (CES-DC) |
| Bele et al. (2012) | Strength and Difficult Questionnaire (SDQ). Maternal depression by Patient Health Questionnaire (PHQ-9), and semi-structured questionnaire |
| Betancourt et al. (2016) | Questionnaire and interview |
| Burlaka et al.(46) | Face to face interview. The Internalising Problems of the Youth Self-Report, Items from the Drinking and Drug History and Current Use Patterns Questionnaire, Alabama Parenting Questionnaire (APQ), and maternal depression by Center for Epidemiologic Studies Depression Scale (CES-D) |
| Chander et al. (2017) | The Strengths and Difficulties Questionnaire (SDQ), Questions related to intimate partner violence, Alcohol Use disorder Identification tests, PTSD and depression was measured by Client Diagnostic Questionnaire, |
| Ciftci and Arzu (2015) | Semi-structured socioeconomic form, WHO student drug-use questionnaire |
| da Rocha et al. (2006) | Interview using standardised protocol developed by the authors |
| Drabick et al. (2004) | Mother-defined groups were created using the Child Symptom Inventory-4 (CSI-4). The CSI-4 is a screening instrument for behavioural symptoms of most DSM-IV childhood disorders. |
| Jardin et al. (2016) | AUDIT form. The Strength and difficulty questionnaire (SDQ) was completed by children, teachers and care-giver |
| Jogdand et al. (2014) | Checklist proforma (adapted from Child Behaviours checklist, CBCL, and Achenbach system of empirically based  assessment behaviour problem check list) |
| Kheokao et al. (2013) | Cross-sectional survey and focus groups; Alcohol Use Disorder Identification Test (AUDIT) |
| Kilic and Sener (2005) | Structured interview, CBCL; McMaster Family Assessment Device (FAD) |
| Krishnakumar et al. (2011) | National Institute of Mental Health Diagnostic Interview Schedule for Children |
| Mansharamani et al. (2018) | A semi-structured sociodemographic proforma and the Child Psychopathology Measurement Schedule (CPMS) |
| Meyer et al. (2013) | Free list interview and key informant interview |
| Narang et al. (1997) | Childhood psychopathology measurement schedule (CPMS); temperament measurement schedule |
| Nothling et al. (2013) | Mothers condition was assessed by the Life Events Checklist (LEC), Harvard Trauma Scale (HTS), Alcohol Use Disorders Identification Test (AUDIT), Center for Epidemiological Studies Depression (CESD) scale and the Sheehan Disability Scale (SDS). Child behaviour was assessed at 42 months with the Child Behaviour checklist (CBCL) |
| Pajarn et al. (2012) | Strength and Difficulty questionnaire (SDQ), Alcohol Use Disorder Identification Test (AUDIT) for parental hazardous and harmful alcohol consumption pattern |
| Pillay and van der Veen (1997) | examination of case records |
| Rahi et al. (2005) | Structured interview and Childhood Psychopathology Measurement schedule (CPMS) |
| Raman et al. (2010) | Questionnaire (“Malin’s Intelligence Scale for Indian Children (MISIC), Child Behaviour Checklist (CBCL), Trail Making Test, Neurodevelopment Scale and the Family Environment Scale”, Severity of Alcohol Dependence Questionnaire SADQ, GHQ questionnaire for psychopathology), Family Environment Scale (FES) |
| Rochat et al. (2019) | KABC learning scale, Child Behaviour Checklist (CBCL), AUDIT, PHQ-9, GAD-7, Parenting Stress Index |
| Ruchkin et al. (2008) | Child behaviour checklist (CBCL) |
| Shenoy et al. (1996) | Scholastic backwardness was operationally defined as poor overall academic performance. Children’s Behaviour Questionnaire (CBQ) |
| Wood et al. | Questionnaire designed by authors |
| Xing et al. (2010) | Questionnaire designed by authors. Suicide attempt was defined according to Youth Risk Behaviour Survey. |
| Yang and Kramer (2012) | Interview and questionnaire. Wechsler Abbreviated Scales of Intelligence (WASI).  SDQ questionnaire filled out by teachers |
| Zanoti-Jerony et al. (2005) | Questionnaire (Rutter A2 Scale of Behaviour of children, The Piers-Harris Children's Self-Concept Scale) |

**Search terms for MEDLINE: Supplementary File 2**

Term 1: Alcohol use

(alcohol us* OR alcohol abus* OR alcohol drink* OR alcohol drinking OR alcohol consum* OR binge drink* OR heavy drink* OR moderate drinking OR drinking behavior* OR Drinking problem* OR ethanol OR ethanol consum* OR ethanol abus* OR ethanol us* OR alcoholism OR alcoholic OR alcoholic beverage* OR alcohol exposure OR ethanol exposure OR alcohol dependen* OR ethanol dependen* OR AUDIT OR CAGE).mp

Term 2: Household

(Household OR family OR family environment OR family histor* OR parent* OR mother OR father OR carer* OR primary caregiver OR caregiver OR caregiver* OR relative$ OR cohabitant$ OR grandmother OR grandfather).mp

Term 3: Young Person

(Child* OR Children OR young children OR young child OR young boy* OR young girl* OR youngster* OR childhood OR early childhood OR late childhood OR infant* OR toddler OR adolescent$ OR youth* OR teen OR teenager* OR preadolescence OR pre-adolescence OR preadolescent OR pre-adolescent OR boy* OR girl* OR son* OR daughter* OR minor* OR juvenile* OR newborn OR schoolboy* OR schoolgirl* OR offspring* OR kindergarten).mp

Term 4: Neurodevelopmental outcome(s)

(development* OR child development* delay* OR childhood disability OR child disability IQ OR intelligence quotient OR intelligence test* OR learning disabilit* OR learning disorder* OR developmental disability* OR psychological OR psychological disorder* OR developmental OR developmental disorder* OR communication OR communicat* disorder* OR articulation disorder* OR Neurodevelopmental disorder* OR language delay* OR communication disorder* OR communication development* disorder OR communicat* dysfunction* OR behavior problem$ OR behavior difficulties OR Acquired language disability OR speech delay* OR language delay* OR speech disorder* OR language disorder* OR mental health OR social behavior$ OR abnormal behavior$ OR under-nutrition OR neurodevelopment* OR cognitive problem* OR cognitive delay* OR cognitive development OR delinquen* OR suicid* OR truan* OR disobedien* OR destructive OR teen pregnancy OR expulsion OR Conduct disorder* OR Oppositional defiant disorder* OR Disruptive behaviour* OR Disruptive behaviour* OR Disruptive behavior disorder* OR disruptive behaviour disorder* OR absenteeism OR lesson disrupt* OR class disrupt* OR risk* sexual behavior* OR risk* sexual behaviour* OR school failure OR drop-out OR absenteeism OR pregnancy).mp
